# Evaluation of the P Wave Axis in Patients With Systemic Lupus Erythematosus

**DOI:** 10.15171/jcvtr.2015.33

**Published:** 2015-11-26

**Authors:** Rezzan Deniz Acar, Mustafa Bulut, Şencan Acar, Servet Izci, Serdar Fidan, Mahmut Yesin, Suleyman Cagan Efe

**Affiliations:** ^1^ Kartal Kosuyolu Education and Research Hospital, Department of Cardiology, Istanbul, Turkey; ^2^ Department of Internal Medicine, Bilim University, Istanbul, Turkey

**Keywords:** P Wave Axis, Systemic Lupus Erythematosus, Left Atrial Volume, Left Atrial Strain

## Abstract

***Introduction:*** P wave axis is one of the most practical clinical tool for evaluation of cardiovascular disease. The aim of our study was to evaluate the P wave axis in electrocardiogram (ECG), left atrial function and association between the disease activity score in patients with systemic lupus erythematosus (SLE).

***Methods:*** Standard 12-lead surface ECGs were recorded by at a paper speed of 25 m/s and an amplifier gain of 10 mm/mV. The heart rate (HR), the duration of PR, QRS, QTd (dispersion), the axis of P wave were measured by ECG machine automatically.

***Results:*** The P wave axis was significantly increased in patients with SLE (49 ± 20 vs. 40 ± 18, *P* = 0.037) and the disease activity score was found positively correlated with P wave axis (r: 0.382, *P* = 0.011). The LA volume and the peak systolic strain of the left atrium (LA) were statistically different between the groups (*P* = 0.024 and P = 0.000). The parameters of the diastolic function; E/A and E/e’ were better in the control group than the patients with SLE (1.1 ± 0.3 vs. 1.3 ± 0.3, *P* = 0.041 and 6.6 ± 2.8 vs. 5.4 ± 1.4, *P* = 0.036, respectively).

***Conclusion:*** P wave axis was found significantly increased in patients with SLE and positively correlated with SELENA-SLEDAI score. As the risk score increases in patients with SLE, P wave axis changes which may predict the risk of all-cause and cardiovascular mortality.

## Introduction

The arrhythmias have higher incidence in patients with systemic lupus erythematosus (SLE) than in the general population due to the rhythm and conduction disturbances.^1^ The major causes of the dysfunction of sinus or AV nodes in SLE are the small vessel vasculitis and the infiltration by fibrous or granulation tissue. Sinus tachycardia, atrial fibrillation and atrial ectopic beats are most frequent and transient supraventricular arrhythmias which may be related to myocarditis and exacerbations of SLE.^2^

P wave axis is one of the routinely reported variables on the printout of most electrocardiograms (ECGs) that may be the most practical clinical tool for evaluation of cardiovascular disease and may provide better predictive value than the currently used P wave indexes. The P wave is the first positive deflection on the ECG and represents atrial depolarisation. Normal P wave axis is between 0° and +75°. However, the relationship between P wave abnormalities and the cardiovascular death has been determined before.^3^

The left atrium (LA) size was found that it largely influences diastolic LV filling which reflects the duration and severity of the diastolic dysfunction.^4^ The previous studies demonstrate that the advanced diastolic dysfunction is strongly associated with increased mortality.^5-7^

The aim of our study is to evaluate the P wave axis in ECG, left atrial function and association between the disease activity score in patients with SLE.

## Materials and Methods

### Study Protocol

We planned a study with 43 experimental subjects and 32 control subjects by using the PS Power and Sample Size Program. In a previous study the response within each group was normally distributed and the population means of the experimental and control groups were equal with probability (power) 0.803. The Type I error probability associated with this test of null hypothesis was 0.05.

The experimental subjects fulfilled at least four of the American College of Rheumatology criteria for SLE.^8^ The screening for coronary artery diseases was undertaken in all cases. Among patients with SLE included in the study, 38 patients had normal cardiac stress test, 3 patients had normal myocardial perfusion SPECT and 2 patients had normal coronary angiogram. Patients with known coronary artery disease, left bundle branch block in ECG, arrhythmia, pericarditis, pulmonary hypertension, congestive heart failure, stroke and peripheral arterial disease were excluded. Also, none of the patients had a history of dissection, any previous cardiac surgery or any clinical disorders known to compromise myocardial function such as diabetes mellitus, renal impairment, anemia, thyroid or liver disorder. In addition, smoking and excessive alcohol consumption were considered as exclusion criteria. We also excluded the patients with other forms of autoimmune diseases. SLE disease activity was assessed using the SELENA-SLEDAI score.^9,10^ It was evaluated by the past history, physical examination, laboratory findings and consultation with their rheumatologists.

The control group consisted of 32 individuals without significant differences in age, sex, weight or height from the patients with SLE. The control group was evaluated carefully and the individuals with the similar demographic characteristics were enrolled the study.

### Electrocardiography

Standard 12-lead surface ECGs were recorded by ‘The Cardiac Science Burdick Atria 6100 EKG Machine’ at a paper speed of 25 m/s and an amplifier gain of 10 mm/mV. The heart rate (HR), the duration of PR, QRS, QTd (dispersion), the axis of P wave were measured by ECG machine automatically.

### Echocardiography

Two-dimensional grayscale harmonic images at a frame rate of 60 to 80 frames/s were obtained in the left lateral decubitus position using a commercially available system (Vivid 7, GE, Horten, Norway). The LV ejection fraction was calculated by Simpson's biplane method of discs according to the American Society of Echocardiography.^11^ Pulsed-wave Doppler at the tip of mitral valve leaflets allowed us to measure the early (E) and late (A) diastolic filling velocities and we calculated the E/A ratio. Also, the peak early diastolic myocardial tissue velocity was measured by tissue Doppler imaging of the lateral mitral annulus (e’) and E/e' was calculated. The diameters of the LA were measured; the maximum anterio-posterior diameter (D1), superior-inferior diameter (D2) and medial-lateral diameter (D3). LA volume was calculated by the formula; D1×D2×D3×0.523. To measure the peak systolic longitidunal left atrial strain by 2-dimensional speckle tracking echocardiography, the gray scale image of apical 4-chamber and 2-chamber views were obtained with the frame rates of 50-80 Hz. The process was recorded with software (EchoPAC, GE Healthcare, Horten, Norway), allowing off-line analyses.

### Statistical Analysis

Statistical analyses were performed using SPSS software version 15.0 (SPSS Inc., Chicago, IL). Data are presented as mean ± standard deviation for continuous variables and as proportions for categorical variables. For all statistical analyses, a two-tailed *P *< 0.05 was considered significant. Simple correlations were evaluated by Pearson’s r correlation coefficients. Independent samples *t* test was used to test differences between groups.

## Results

Of the 43 patients with SLE enrolled in this study, 8 were male (18%). Demographic properties and echocardiographic parameters of both the SLE patients and the control group are shown in Table 1.

**Table 1:** Demographic Properties, Clinical Characteristics of Both the SLE Patients and the Control Group

	**SLE patients (n=43)** **(Mean ± SD)**	**Control Group (n=32)** **(Mean ± SD)**	*** P *** ** Value**
Age (y)	35.7 ± 13.7	35.9 ± 10.0	0.962
Gender (n)	8 male (18 %)	7 male (21 %)	0.519
Weight (kg)	70.2 ± 11.7	76.5 ± 9.6	0.052
Height (cm)	163 ± 8.0	166 ± 7.7	0.106
Hypertension (n)	11 (25 %)	9 (28 %)	0.682

Abbreviations: n, number; SD, standart deviation; SLE, systemic lupus erythematosus.

**Figure 1:**
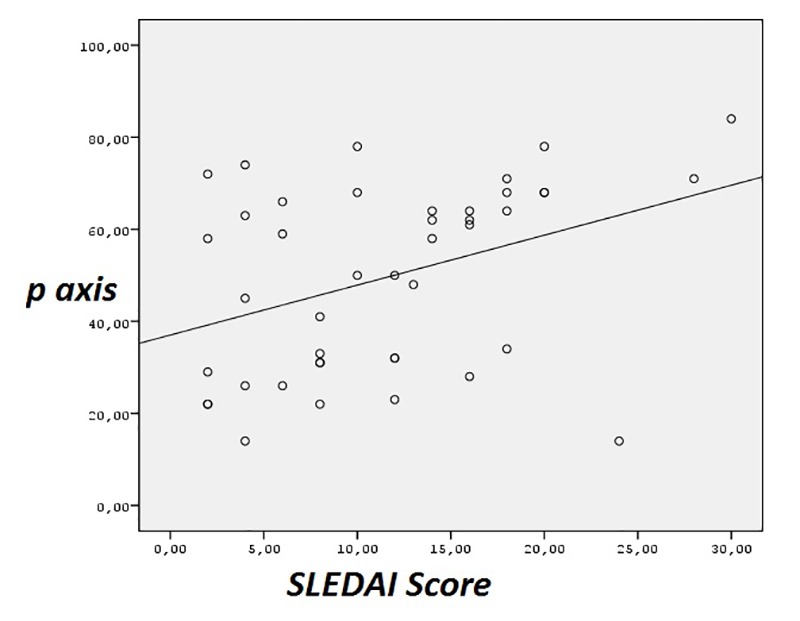
The Positive Correlation Between the P Wave Axis and the SLE Disease Activity Score (SELENA-SLEDAI). (r = 0.382, P = 0.011).

The HR, the duration of PR, QRS, and the axis of QRS and T wave were not statistically significant. The P wave axis was significantly changed in patients with SLE (49 ± 20 vs. 40 ± 18, *P *= 0.037) and the disease activity score was found positively correlated with P wave axis (r = 0.382, *P *= 0.011, Figure 1) However, the QTd was significantly increased in patients with SLE (42 ± 21 vs.33 ± 16, *P *= 0.046; Table 2)

**Table 2:** Evaluation of the ECG Parameters of Both the SLE Patients and the Control Group

	** SLE patients (n = 43) ** ** (Mean ± SD) **	** Control Group (n = 32) ** ** (Mean ± SD) **	*** P *** ** Value**
HR (per min)	73 ± 10	76 ± 10	0.130
PR (msec)	148 ± 18	144 ± 23	0.338
QRS (msec)	85 ± 7.4	87 ± 10	0.422
QTd (msec)	42 ± 21	33 ± 16	0.046
P axis (degree)	49 ± 20	40 ± 18	0.037
QRS axis (degree)	40 ± 34	40 ± 25	0.962
T axis (degree)	41 ± 20	40 ± 15	0.824

Abbreviations: n, number; SD, standart deviation; SLE, systemic lupus erythematosus; HR, heart rate.

The LA volume and the peak systolic strain of the LA were statistically different between the groups (*P *= 0.024 and *P *= 0.000). The left ventricular end diastolic volume (LVED volume) and ejection fraction (EF) of the patients with SLE and the control group were similar (93.8 ± 20.7 vs. 91.6 ± 20.8, *P *= 0.659, 60.9 ± 10 vs. 64.7 ± 5.5, *P *= 0.073, respectively). The Sm lateral and Sm septal with tissue Doppler echocardiography were significantly lower in SLE (9.4 ± 2.0 vs. 12.3 ± 2.2, *P *= 0.000 and 8.1 ± 1.3 vs. 10.4 ± 1.4, *P *= 0.000, respectively). Also, the parameters of the diastolic function; E/A and E/e’ were better in the control group than the patients with SLE (1.1 ± 0.3 vs.1.3 ± 0.3, *P *= 0.041 and 6.6 ± 2.8 vs. 5.4 ± 1.4, *P *= 0.036, respectively) (Table 3).

**Table 3:** The Echocardiographic Parameters of Both the SLE Patients and the Control Group

	**SLE Patients (n=43) ** **(Mean ± SD)**	**Control Group (n=32)** ** (Mean ± SD)**	*** P *** **Value**
LVd (cm)	4.7 ± 0.44	4.5± 0.36	0.196
LVs (cm)	2.7 ± 0.35	2.5 ± 0.36	0.128
IVS (cm)	0.90 ± 0.12	0.91 ± 0.12	0.801
Posterior wall (cm)	0.79 ± 0.12	0.78 ± 0.11	0.629
LVED volume (ml)	93.8 ± 20.7	91.6 ± 20.8	0.659
EF %	60.9 ± 10	64.7 ± 5.5	0.073
E/A	1.1 ± 0.3	1.3 ± 0.3	0.041
E/e’	6.6 ± 2.8	5.4 ± 1.4	0.036
LA volume	30 ± 4.3	28 ± 4.2	0.024
LA strain	19.2 ± 1.5	20.9 ± 1.5	0.000
Sm lateral	9.4 ± 2.0	12.3 ± 2.2	0.000
Sm septal	8.1 ± 1.3	10.4 ± 1.4	0.000

Abbreviations: n, number; SD, standard deviation; SLE, systemic lupus erythematosus; LVd, left ventricle diastolic diameter; LVs, left ventricle systolic diameter; IVS, interventricular septum; LVED, left ventricle end-diastolic diameter; EF, ejection fraction; LA, left atrium; Sm: peak systolic velocity

## Discussion

P wave axis was found significantly changed and positively correlated with SELENA-SLEDAI score in patients with SLE, in this study. The determination of P wave axis is important because in the analysis from the NHANES III survey, abnormal P wave axis was found associated with an increased risk of all-cause and cardiovascular mortality in a representative sample from the US population.^12^

The principal role of the LA is to modulate and augment the left ventricular filling trough the late diastole like a booster pump. An emerged parameter in assessing LA function is longitudinal strain which is a robust, well validated and reproducible technique for the measurement of LA longitudinal deformation. The impairment of left atrial function has many serious consequences. Sasaki et al has reported that decreased LA peak systolic strain was also independently associated with LA appendage dysfunction in patients with acute ischemic stroke.^13^ Li et al recently demonstrated that according to the Systemic Lupus International Collaborating Clinics/American College of Rheumatology Damage Index (SDI), patients with a SDI ≥ 1, left atrial mechanical function and volume were found impaired in SLE patients with an increased disease activity. They reported the impairment in left atrial performance by using real-time 3-D echocardiography (RT3DE) technology.^14^ We found significantly decreased values of the left atrial peak systolic strain by using 2–dimensional speckle tracking echocardiography and larger volumes of the LA than the control group. Kurt et al were demonstrated that diastolic heart failure is associated with larger LA volumes and decreased LA peak systolic strain.^15^ The major problem caused an increase in LA volume and decrease in LA peak systolic strain in patients with SLE may be the reduced diastolic reserve due to the abnormal myocardial relaxation.

According to a study by Win et al, there was an association between p wave duration and increase in LA volume and decrease in LA emptying fraction and reservoir function but they did not find any association with P wave axis.^16^ Likewise, we could not find any association between the P wave axis and the LA volume and LA peak systolic strain.

Also, increased QTd can be seen because of myocardial involvement even in the absence of clinical cardiac manifestations in patients either with SLE and other rheumatologic disorders such as rheumatoid arthritis and ankylosing spondylitis.^17-20^ In our study, QTd which predicts the increased risk of ventricular tachyarrhythmias was found significantly increased in patients with SLE alike the literature.

SLE disease activity score was found positively correlated with the P wave axis degrees which means that; as the SLE activity getting impaired, P wave axis degree increases that may also increase the risk of all-cause and cardiovascular mortality in patients with SLE.

## Study Limitations

First of all, the number of our patients is few. Second, the long term monitoring to detect the possible arrhythmia or the risk of mortality is lacking. Altough SLE patients did not have abnormal P wave axis, we should emphasize that they were in the remission period. The P wave axis degrees are greater than the control group even in the lack of disease activity. The most important limitation of our study is, we did not compare them with the SLE patients in the active period.

## Conclusion

P wave axis was found significantly changed in patients with SLE and positively correlated with SELENA-SLEDAI score. The LA volume was increased and the LA peak systolic strain was decreased in patients with SLE, but there was not any association with the P wave axis. Consequently, as the risk score increases in SLE, P wave axis changes which may predict the risk of all-cause and cardiovascular mortality.

## Ethical issues

Written informed consent was obtained from each subject, and the institutional ethics committee approved the study protocol.

## Competing interests

Authors declare no conflict of interest in this study.

## References

[1] Teixeira RA, Borba EF, Bonfá E, Martinelli Filho M (2010). Arrhythmias in systemic lupus erythematosus. Rev Bras Reumatol.

[2] Abu-Shakra M, Urowitz MB, Gladman DD, Gough J (1995). Mortality studies in systemic lupus erythematosus: results from a single center I Causes of death. J Rheumatol.

[3] Kaykha A, Myers J, Desser KB, Laufer N, Froelicher VF (2010). The prognostic importance of isolated P-Wave abnormalities. Clin Cardiol.

[4] Simek CL, Feldman MD, Haber HL, Wu CC, Jayaweera AR, Kaul S (1995). Relationship between left ventricular wall thickness and left atrial size: comparison with other measures of diastolic function. J Am Soc Echocardiogr.

[5] Nijland F, Kamp O, Karreman AJ, van Eenige MJ, Visser CA (1997). Prognostic implications of restrictive left ventricular filling in acute myocardial infarction. J Am Coll Cardiol.

[6] Moller JE, Sondergaard E, Poulsen SH, Egstrup K (2000). Pseudonormal and restrictive filling patterns predict left ventricular dilation and cardiac death after a first myocardial infarction. J Am Coll Cardiol.

[7] Cerisano G, Bolognese L, Buonamici P, Valenti R, Carrabba N, Dovellini EV (2001). Prognostic implications of restrictive left ventricular filling in reperfused anterior acute myocardial infarction. J Am Coll Cardiol.

[8] Hochberg MC (1997). Updating the American College of Rheumatology revised criteria for the classification of systemic lupus erythematosus. Arthritis Rheumatol.

[9] Bombardier C, Gladman DD, Urowitz MB, Caron D, Chang CH (1992). Derivation of the SLEDAI A disease activity index for lupus patients The Committee on Prognosis Studies in SLE. Arthritis Rheum.

[10] Buyon JP, Petri MA, Kim MY, Kalunian KC, Grossman J, Hahn BH (2005). The effect of combined estrogen and progesterone hormone replacement therapy on disease activity in systemic lupus erythematosus: a randomized trial. Ann Intern Med.

[11] Lang RM, Bierig M, Devereux RB, Flachskampf FA, Foster E, Pellikka PA (2005). American Society of Echocardiography's Guidelines and Standards Committee; European Association of Echocardiography Recommendations for Chamber Quantification: A Report from the American Society of Echocardiography’s Guidelines and Standards Committee and the Chamber Quantification Writing Group, Developed in Conjunction with the European Association of Echocardiography, a Branch of the European Society of Cardiology. J Am Soc Echocardiogr.

[12] Li Y, Shah AJ, Soliman EZ (2014). Effect of electrocardiographic P-wave axis on mortality. Am J Cardiol.

[13] Sasaki S, Watanabe T, Tamura H, Nishiyama S, Wanezaki M, Sato C (2014). Left atrial strain as evaluated by two-dimensional speckle tracking predicts left atrial appendage dysfunction in patients with acute ischemic stroke. BBA Clin.

[14] Li K, Wang R, Dai M, Lu J, Zou Y, Yang X (2015). Evaluation of left atrial function by real-time 3-D echocardiography in patients with systemic lupus erythematosus. J Rheumatol.

[15] Kurt M, Wang J, Torre-Amione G, Nagueh SF (2009). Left atrial function in diastolic heart failure. Circ Cardiovasc Imaging.

[16] Tiffany Win T, Ambale Venkatesh B, Volpe GJ, Mewton N, Rizzi P, Sharma RK (2015). Associations of electrocardiographic P-wave characteristics with left atrial function, and diffuse left ventricular fibrosis defined by cardiac magnetic resonance: The PRIMERI Study. Heart Rhythm.

[17] Yildirir A, Aksoyek S, Calguneri M, Aytemir K, Kabakci G, Ovunc K (2000). QT dispersion as a predictor of arrhythmic events in patients with ankylosing spondylitis. Rheumatology.

[18] Pirildar T, Sekuri C, Uyuk O, Kemal Tezcan U (2003). QT dispersion in rheumatoid arthritis patients with and without Sjogren’s syndrome. Clin Rheumatol.

[19] Cindas A, Gokce-Kutsal Y, Tokgozoglu L, Karanfil A (2002). QT dispersion and cardiac involvement in patients with rheumatoid arthritis. Scand J Rheumatol.

[20] Yavuz B, Atalar E, Karadag O, Tulumen E, Ozer N, Akdogan A (2007). QT dispersion increases in patients with Systemic lupus erythematosus. Clin Rheumatol.

